# Meta-analysis of the effects of multi-component exercise on cognitive function in older adults with cognitive impairment

**DOI:** 10.3389/fnagi.2025.1551877

**Published:** 2025-04-14

**Authors:** Hualei Liu, Zhiyuan Sun, Haiqing Zeng, Jincheng Han, Mengqi Hu, Dewei Mao, Xuewen Tian, Ran Li

**Affiliations:** ^1^Shandong Sports Science Research Institute, Shandong Sports University, Jinan, Shandong, China; ^2^Qufu Normal University, Jining, Shandong, China; ^3^The Chinese University of Hong Kong, Shenzhen, China

**Keywords:** multi-component exercise, elderly, cognitive function, meta-analysis, exercise

## Abstract

Exercise has been widely recognized as an effective regimen in mitigating cognitive decline. However, the effect of multi-component exercise (i.e., combination of two or more types of exercise) on cognitive function and its subdomains in older adults remains unclear. This meta-analysis aimed to explore the effects of multi-component exercise on cognitive functions in elderly individuals with cognitive impairment and identify optimal prevention and treatment strategies. A systematic search was conducted on PubMed, EBSCOhost, Web of Science, and Embase to identify relevant randomized controlled trials assessing the effect of multi-component exercise on cognitive function in the elderly. Thirteen studies with 1,776 participants were included in the analysis using Revman 5.4 software. The results showed that multi-component exercise had a significant effect on mitigating cognitive function decline in the elderly, with a pooled effect size of SMD = 0.31 (95% CI: 0.08, 0.55; *p* = 0.009). The results of subgroup analysis showed that interventions with ≥3 days/week, 12–24 weeks duration, and ≤ 40 min/session were significantly superior to other frequencies, durations, and lengths, with all *p*-values <0.05. Additionally, multi-component exercise had the most pronounced effects on executive function, visual memory, and verbal memory in patients with mild cognitive impairment (MCI). In conclusion, multi-component exercise can delay the decline in cognitive function in the elderly, and the intervention effects are modulated by various variables. Optimal intervention effects were observed with an exercise frequency of three or more times per week, a duration of 12 to 24 weeks, and a time per session of 40 min or less, particularly for improving executive function, visual memory, and verbal memory in patients with MCI.

## 1 Introduction

As the global population ages at an accelerated pace, the prevalence of aging-related chronic diseases is on the rise, presenting numerous challenges for health organizations worldwide (Tabloski, 2004). According to the latest report from the World Health Organization (WHO), around 50 million elderly individuals globally are grappling with cognitive dysfunction (Rudnicka et al., 2020). Cognitive decline in the elderly encompasses normal age-related cognitive changes, subjective cognitive impairment (complaints about cognitive function on standard screening tests), MCI, and dementia (Jongsiriyanyong and Limpawattana, 2018). Cognitive dysfunction serves as an early indicator of Alzheimer’s disease (AD), impacting key cognitive domains such as learning and memory, social interactions, language skills, visuospatial abilities, attention span, and executive functions. The onset of cognitive impairment can trigger other neurological, psychological, or systemic conditions, significantly compromising the independence and quality of life of older adults and imposing a substantial societal burden. Hence, the prevention and early detection of cognitive dysfunction in the elderly are crucial.

There is currently insufficient evidence to support the effectiveness of medication in treating cognitive impairment (Tisher and Salardini, 2019), especially in the early stages of its development. As a result, non-drug treatment for cognitive impairment have gained significant attention in research (Shah et al., 2016). Physical activity and exercise, as key components of non-drug treatments, have shown positive effects on enhancing cognitive function. Various systematic reviews and meta-analyses have indicated that physical exercise may help improve cognitive function in individuals with MCI (Zheng et al., 2016; Du et al., 2018; Song et al., 2018). Research suggests that multi-component exercise, incorporating different types of aerobic, resistance, balance, and flexibility training (Cress et al., 2005), is more effective in enhancing cognitive function compared to single exercises (Vaughan et al., 2014). For instance, a study by Espeland et al. (2017) implemented a multi-component exercise program involving aerobic, resistance, balance, and flexibility training in sedentary older adults aged 70–89 without cognitive impairment, showing significant improvements in overall cognitive function and delayed memory in elderly individuals with diabetes. The beneficial impact of exercise on cognitive function may be attributed to mechanisms such as enhancing glucose control and improving vascular function. Another study by Cordes et al. (2019) conducted a 16-week intervention in older adults with mild MCI in nursing homes, utilizing various coordinated balance, walking, strength training, and flexibility exercises, further supporting the positive effects of multi-component movements on cognitive function. The findings indicate that the multi-component exercise intervention was effective in enhancing gait speed, balance ability, cognitive performance, and quality of life in the elderly, while also encouraging social engagement. Additionally, multi-component exercise is recommended for elderly patients experiencing functional decline, frailty, and sarcopenia during hospitalization (Sadjapong et al., 2020).

Venegas-Sanabria et al. (2022) conducted a meta-analysis revealing that only multi-component exercise that includes aerobic exercise has a noteworthy effect on enhancing overall cognitive ability. Contrarily, Blomstrand et al. (2023) found that mind-body exercise has a more substantial impact on cognitive function in healthy adults over 55 years old compared to aerobic exercise. Wang et al. (2020) conducted a meta-analysis and the authors suggested that multi-component exercise led to significant enhancements in cognitive ability, attention, and executive functions, but did not significantly affect memory. However, a randomized controlled trial demonstrated that the group undergoing aerobic exercise combined with strength multi-component training experienced notable improvements in overall cognition, visual memory, verbal memory, and executive function compared to the social activity control group (Bossers et al., 2015). These conflicting findings highlight the need for further validation through additional meta-analyses.

Current research on the effects of various types of multi-component exercise intervention on cognitive impairment and the specific cognitive subdomains it targets remains inconclusive. Previous studies have not specifically analyzed key parameters of exercise intervention, such as time, frequency, and period, and the types of diseases in the subjects. To accurately assess the influence of multi-component exercise on elderly cognitive function, intervention duration, period, and frequency must be carefully considered across studies. In light of this, our systematic review and meta-analysis aim to investigate the influence of multi-component exercise on overall cognitive function and specific cognitive subdomains in elderly individuals with cognitive impairment. Furthermore, our study seeks to elucidate how different types of cognitive impairment respond to multi-component training and determine the optimal intervention parameters for enhancing cognitive function. By identifying the optimal duration, frequency, and timing of multi-component exercise interventions, we aim to provide solid evidence for clinical practice in this field.

## 2 Methods

This study was conducted in compliance with the Cochrane Reporting Manual for Systematic Reviews and Meta-Analysis and the PRISMA (Preferred Reporting Items for Systematic Reviews and Meta-Analysis) statement (Moher et al., 2009). The registration number for this study is CRD42024533463.

### 2.1 Literature search

Two researchers systematically searched four databases, namely PubMed, EBSCO host, Web of Science, and Embase, to identify all clinical randomized controlled trial studies published in English until January 25, 2024. The search terms used were: Multi-component exercise training, 0multi-mode motion, multi-mode locomotion, multi-modal motion, associated movement, associated movement, multi-locomotion modes; Cognition, MCI, mild neurocognitive disorder, memory disorder, memory impairment, memory decline early-stage dementia, cognitive decline, cognition dysfunction, mental deterioration; senior citizen, elderly and age, aging. The search terms specified combined using Boolean operators ‘OR’ within each group and ‘AND’ between groups in order to retrieve relevant articles. In addition, manual searches were also conducted to follow up on all the references cited in the included documents, thus ensuring the comprehensiveness of the study.

### 2.2 Inclusion and exclusion criteria

We applied the Population, Intervention, Comparison, Outcomes, and Study design (PICOS) framework as the inclusion criteria for this study. Population: older adults aged 60 and above without significant contradictions to exercise and diagnosed with cognitive impairment according to the WHO’s International Classification of Diseases. Interventions: required a combination of two or more exercise modalities (such as aerobic, resistance, cognitive, coordination, flexibility, etc.) with exercise as the sole intervention in the experimental group the control group received either no exercise, routine care, health education, or non-exercise social activities. Comparison: between the intervention group and a control group. Outcomes: average total scores and domain subscale scores from cognitive function assessment tools including the Montreal Cognitive Assessment (MoCA), Mini-Mental State Examination (MMSE), and Alzheimer’s Disease Assessment Scale-Cognitive (ADAS-Cog). Study design: only randomized controlled trails (RCTs) were eligible for inclusion.

Non-relevant papers and duplicate papers were excluded from the study. Additionally, reviews, editorials, animal experiments, theses, and articles lacking complete outcome measures or sufficient data for calculating standardized mean differences (SMD) post-intervention were also excluded.

### 2.3 Study selection and data extraction

The literature search results were imported into the EndNote X9 literature management software for deduplication. Two researchers (LHL and SZY) conducted a double-blind screening of the literature according to the study inclusion and exclusion criteria. Any discrepancies were resolved through discussion or the involvement of a third researcher. Data extraction was carried out independently using a pre-designed form. The following data were extracted for the included studies: (1) the name of the first author and the year of publication; (2) population characteristics, such as sample size, gender ratio, mean age, and cognitive impairment type; (3) intervention characteristics, including intervention content, frequency, time, duration, and intensity of the exercise (if applicable); (4) main outcomes, which involved extracting the average cognitive function assessment tool total scores and domain subscale scores from each study to evaluate cognitive function in older adults.

### 2.4 Quality assessment of the studies

The risk of bias assessment for the included studies was evaluated using the Cochrane Collaboration’s tool for assessing risk of bias in randomized controlled trials (Higgins et al., 2011), which included the following domains: (1) generation of random sequence; (2) allocation concealment; (3) blinding of participants and personnel; (4) blinding of outcome assessment; (5) completeness of outcome data; (6) selective reporting; and (7) other sources of bias. Bias risk assessment was independently conducted by two researchers, and any discrepancies were resolved through discussion or consultation with a third researcher. Each domain was classified as low risk of bias, unclear risk of bias, or high risk of bias. Based on the classification results, the quality of the included studies was divided into three levels: (1) studies meeting four or more criteria with low risk were rated as level A; (2) studies meeting two or three criteria with low risk were rated as level B; (3) studies meeting one or no criteria with low risk were rated as level C.

### 2.5 Statistical analyses

The statistical analysis of outcome measures for the included studies was conducted using Revman 5.4. Heterogeneity testing was initially performed using the Homogeneity test (χ^2^ test) with a significance level of *a* = 0.1. If *P* < a, heterogeneity existed among the studies; otherwise, the studies were considered homogeneous. At the same time, *I^2^* was used to quantify the degree of inconsistency between studies. The thresholds of *I^2^* = 25%, 50, and 75% is an indication of low, moderate, and high heterogeneity, respectively. When meaningful heterogeneity was observed (*I^2^* > 50%), a random effects model of meta-analysis was applied and a sensitivity analysis was conducted. Otherwise, a fixed effect model was used to pool study results.

If there was high heterogeneity among the studies, a subgroup analysis was conducted to identify the sources of heterogeneity. Sensitivity analysis was used to test the stability of the results, and Egger’s test was used to assess publication bias for each outcome measure. A funnel plot was used to analyze publication bias. The effect sizes were calculated using the mean difference for outcome measures with consistent units and SMD for outcome measures with inconsistent units, and a 95% confidence interval was used for analysis. A *p*-value <0.05 was considered statistically significant. The meta-analysis was conducted using the changes in cognition during the entire intervention period and cognitive scores after the intervention for the experimental and control groups.

## 3 Results

### 3.1 Identification of studies

**Figure 1:**
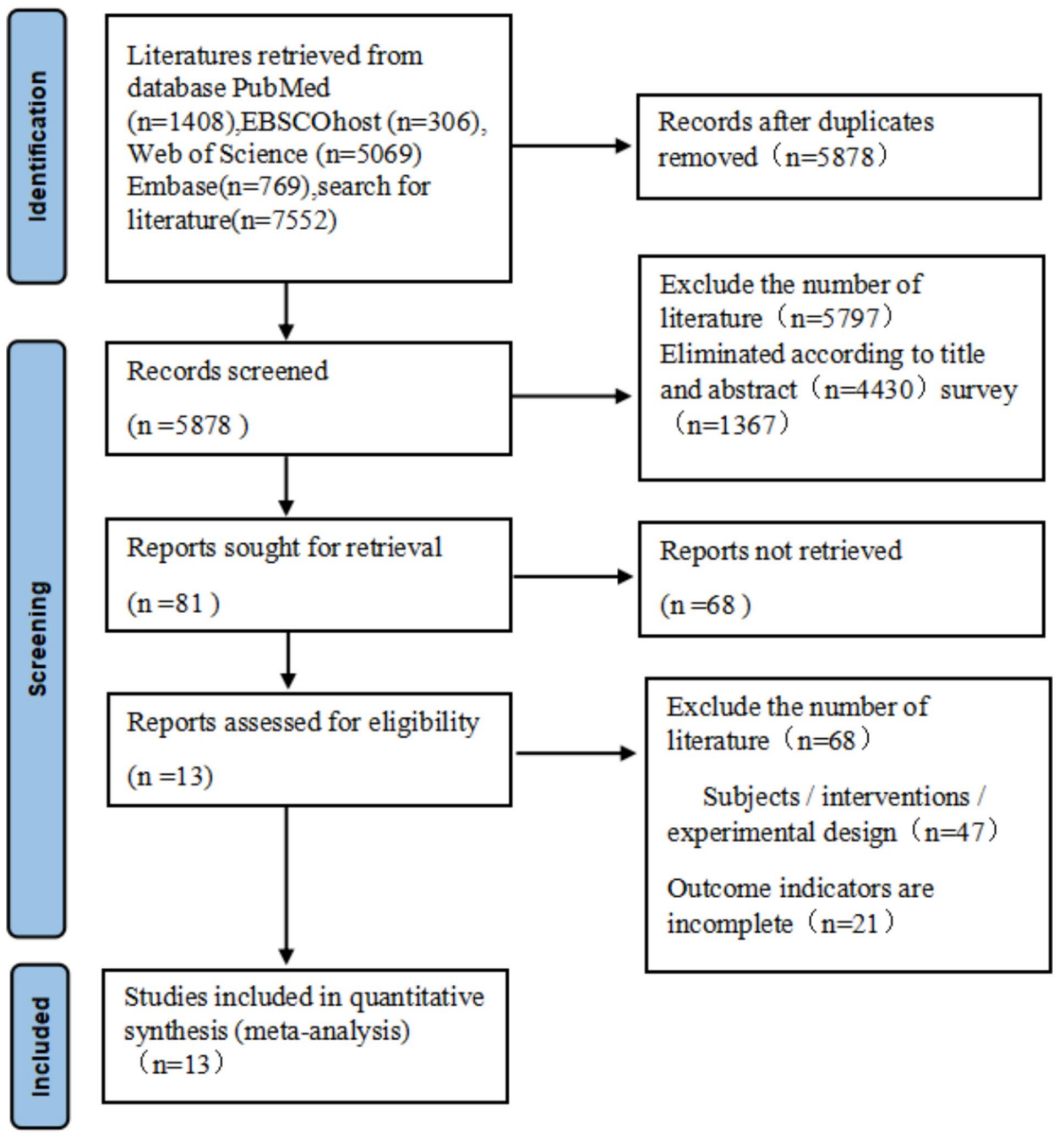
Flow diagram of the study selection procedure.

A total of 7,552 articles were retrieved through electronic databases searching. After removing 1755 duplicated articles, 5,878 articles were considered for abstract reviews. After screening titles and abstracts, 5,797 articles were excluded as unrelated, leaving 81 articles for full-text review. Finally, 13 articles (Bossers et al., 2015; de Souto et al., 2017; Öhman et al., 2016; Vreugdenhil et al., 2012; Lamb et al., 2018; Toots et al., 2017; Langoni et al., 2019; Suzuki et al., 2012; Li et al., 2021; Casas-Herrero et al., 2022; Mak et al., 2022; Makino et al., 2021; Suzuki et al., 2013) met the inclusion criteria to be quantitative analyses. The literature searching process is demonstrated in Figure 1.

### 3.2 Description of included studies

The meta-analysis included 13 studies (total *N* = 1,776; 44% male), with 953 participants in intervention groups and 823 in control groups. Cognitive function was assessed using the MMSE, MoCA, and ADAS-cog across all studies. The populations included Alzheimer’s disease (AD, 2 studies), dementia (5 studies), and mild cognitive impairment (MCI, 6 studies). Intervention parameters varied: frequency (≥3 days/week: 4 studies; <3 days/week: 9 studies), duration (≤12 weeks: 2 studies; 12-24 weeks: 7 studies; >24 weeks: 4 studies), and session time (≤40 min: 4 studies; 40-60 min: 7 studies; >60 min: 2 studies). Control groups received routine care or health education. A comprehensive summary of study characteristics is provided in Table 1.

**Table 1:** Characteristics of the studies included in the qualitative analysis.

First author, year	Subjects				Intervention measures						Result(SMD)
	Sample size	Male /female	Age (years)	Types of cognitive impairment	Exercise description	Frequency (times/week)	Time (min/session)	Duration (week)	Exercise intensity	Therapy for control group	
de Souto et al. (2017)	I:44C:47	15% of Male	I:88.3 ± 5.1 C:86.9 ± 5.8	AD	Aerobic Balance	2	60	24	Moderate	Social Activity Group	MMSE:-0.15(−0.56, 0.26)
Öhman et al. (2016)	I:70C:70	60% of Male	I:77.7 ± 5.4 C:78.1 ± 5.3	AD	Aerobic Strength Balance	2	60	48	Not mentioned	Community care	MMSE:-0.12(−0.46, 0.21)
Bossers et al. (2015)	I:37C:36	26% of Male	I:85.7 ± 5.1 C:85.4 ± 5.0	Dementia	Aerobic Strength	4	30	9	Moderate to high	Social visits	MMSE:0.47(0.01, 0.94)
Vreugdenhil et al. (2012)	I:20C:20	40% of Male	I:73.5 (51–83) C:74.7 (58–89)	Dementia	Aerobic Strength Balance	7	≥30	16	Not mentioned	Usual treatment	MMSE:0.48(−0.15, 1.11)
Lamb et al. (2018)	I:278C:137	61% of Male	I:76.9 ± 7.7 C:78.1 ± 7.7	Dementia	Aerobic Strength	2	60–90	16	Moderate to high	Usual care	ADAS-cog: 0.15(−0.05, 0.36)
Toots et al. (2017)	I:93C:93	24% of Male	I:84.4 ± 6.2 C:85.9 ± 7.8	Dementia	Strength Balance	2–3	45	16	High (defined as 8-12RM)	Attention control activities	MMSE:-0.06(−0.35, 0.23)
Langoni et al. (2019)	I:26C:26	23% of Male	I:72.6 ± 7.8 C:71.9 ± 7.9	MCI	Aerobic Strength	2	60	24	Moderate (60-75%HR_max_)	Not to initiate any kind of physical	MMSE:1.74(1.10, 2.39)
Suzuki et al. (2012)	I:25C:25	54% of Male	I:75.3 ± 7.5 C:76.8 ± 6.8	MCI	Aerobic Strength Balance	2	90	48	Moderate (approximately 60% HR_max_.)	Education control group	MMSE:-0.01(−0.56, 0.54)
Li et al. (2021)	I:42C:42	39% of Male	≥60	MCI	Aerobic Strength Balance	5	30	24	Warm-up: low (3–4/10 RPE) Exercise: moderate (4–5/10 RPE)	General community health instruction	MMSE:1.48(0.99, 1.96)
Casas-Herrero et al. (2022)	I:88C:100	30% of Male	I:84.2 ± 4.8 C:84.0 ± 4.8	Dementia, MCI	Aerobic Strength Balance	3	30	12	Resistance:gradually increase the load until 30 repetitions are reached	Usual-care	MOCA:0.41(0.12, 0.70)
Mak et al. (2022)	I:76C:72	41% of Male	I:86.0 (84.8–87.3) C:87.2 (85.7–88.7)	MCI dementia	Strength Balance	2	60	48	Moderate (12–14/20 RPE)	Usual-care	MMSE:0.16(−0.17, 0.48)
Makino et al. (2021)	I:104C:105	54% of Male	I:72.61 ± 4.52 C:72.10 ± 4.61	MCI	Aerobic Strength	2	60	26	Weeks 1–2: light (11/20 RPE) Weeks 3–12: moderate (13/20 RPE); Weeks 13–26: high (15/20 RPE)	Educational classes	MMSE:-0.03(−0.30, 0.24)
Suzuki et al. (2013)	I:50C:50	51% of Male	I:74.8 ± 7.4 C:75.8 ± 6.1	MCI	Aerobic Strength Balance	2	90	24	Approximately 60% of HR_max_	Education control	MMSE:0.19(−0.21, 0.58)

I, intervention group; C, control group.

### 3.3 Risk of bias

**Figure 2:**
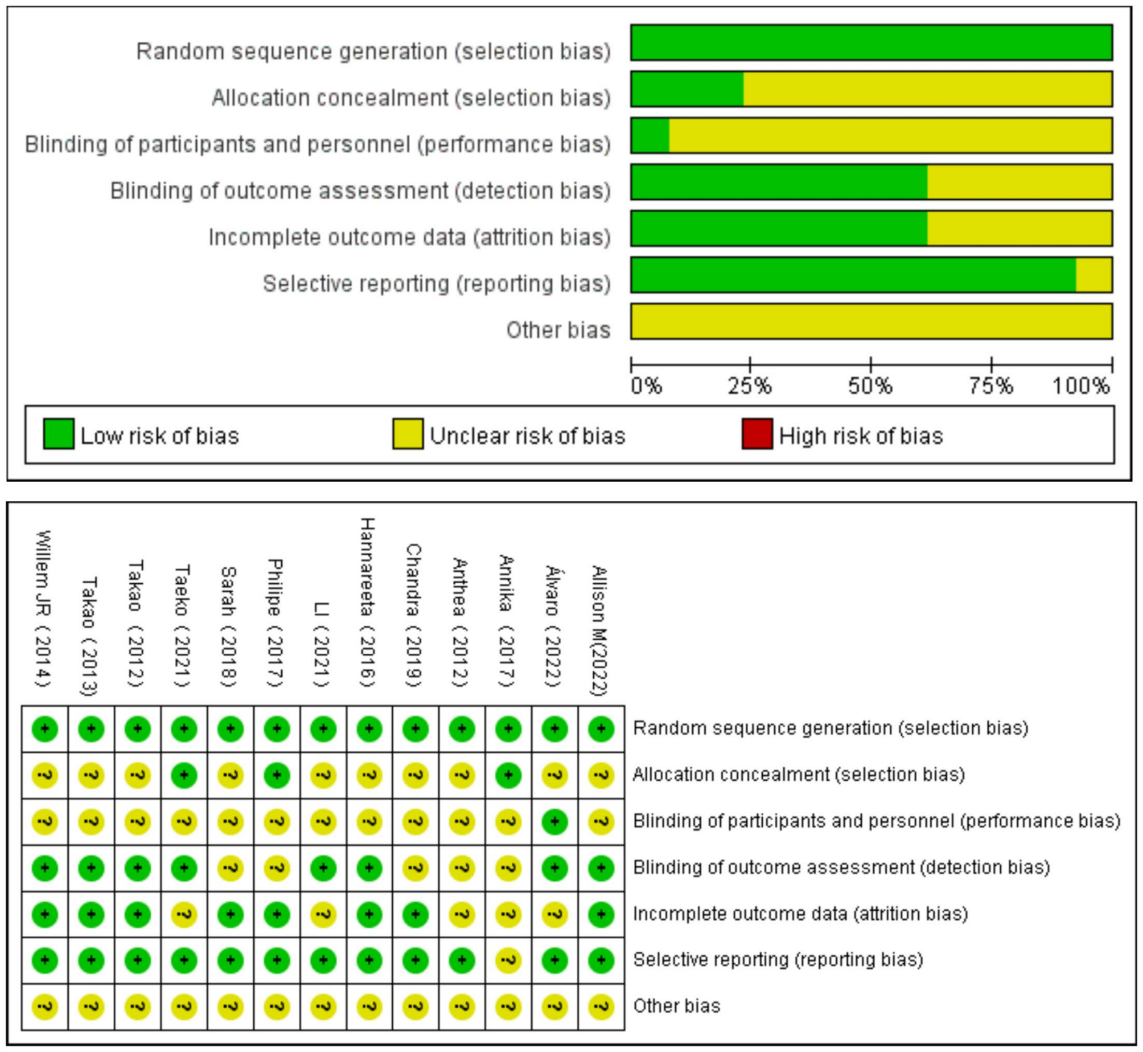
Summary of the risk assessment in the included literature.

The methodological quality of the included studies was assessed using the Cochrane risk of bias assessment tool. As shown in Figure 2, out of the 13 studies included in the analysis, 8 studies met four or more criteria and were assigned an A-level rating for the quality assessment. Five studies met two to three criteria and were assigned a B-level rating for the quality assessment.

### 3.4 Meta-analyses

#### 3.4.1 The effect of multi-component exercise on cognitive functions

**Figure 3:**
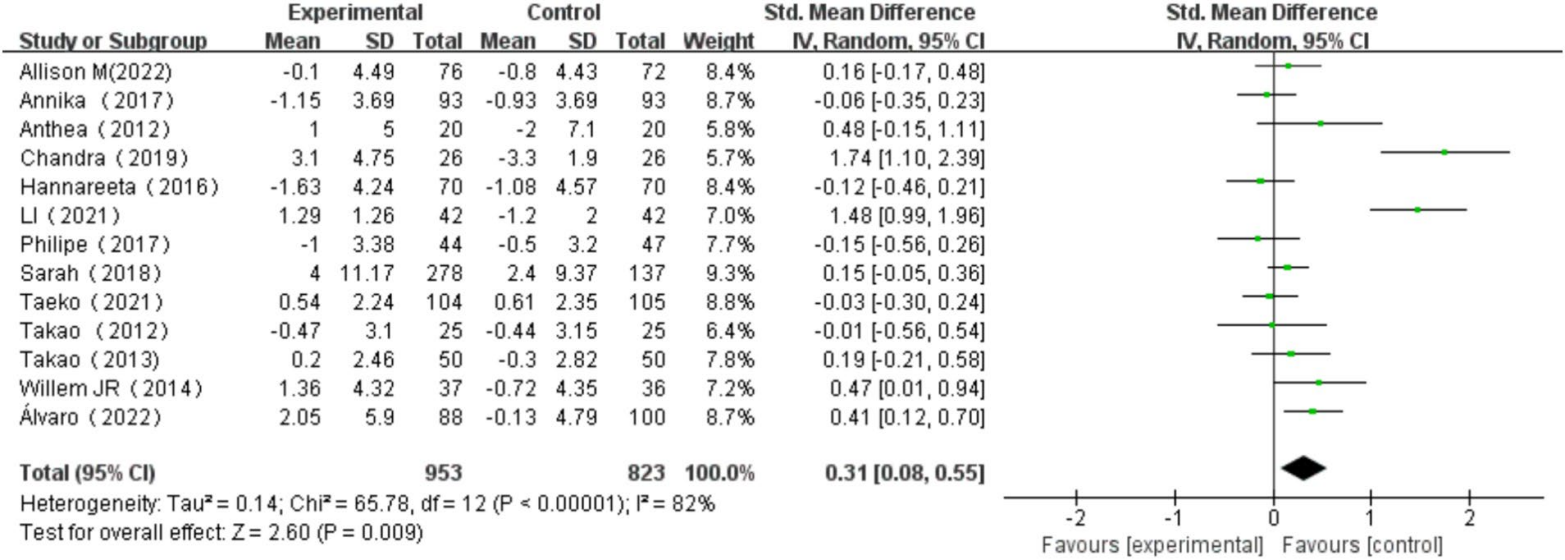
Effect of multi-component exercise on overall cognitive function.

The overall effect of multicomponent exercise on cognitive function in older adults was assessed through comparative analysis of cognitive changes between experimental and control groups during the intervention period across all selected studies. The analysis revealed significant heterogeneity among the studies (*I^2^* = 82% > 50%, *p* < 0.001). Therefore, a random effects model was used to combine the results, yielding a pooled effect size of SMD = 0.31 (95% CI: 0.08, 0.55; *p* = 0.009). The forest plot showed that the 95% CI of the SMD for the impact of multi-component exercise on cognitive function in older adults fell to the right of the line of no effect (see Figure 3), indicating the effectiveness of multi-component exercise in improving cognitive function in older adults.

#### 3.4.2 The effect of multi-component exercise on cognitive subdomains

##### 3.4.2.1 The effect of multi-component exercise on information processing speed

**Figure 4:**
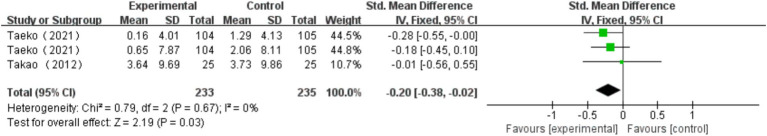
Effect of multi-component exercise on information processing speed in the elderly.

A total of 2 studies (Suzuki et al., 2012; Makino et al., 2021) were included, with one study using two different measurement methods. These measurement methods include Category Fluency Test, WAIS-III Digit Symbol, DSC. Heterogeneity test results showed no significant heterogeneity between the studies (*I^2^* = 0%, *p* = 0.67). A fixed effects model was used to combine the results, yielding a combined effect size of SMD = −0.20, 95% CI: −0.38, −0.02, *p* = 0.03. The forest plot indicated that the impact of multi-component exercise on the cognitive subdomain of information processing speed in older adults had a 95% CI for SMD that fell to the left of the null line (see Figure 4). The results suggest that multi-component exercise has no significant effect on the cognitive subdomain of information processing speed in older adults.

##### 3.4.2.2 The effect of multi-component exercise on executive functions

**Figure 5:**
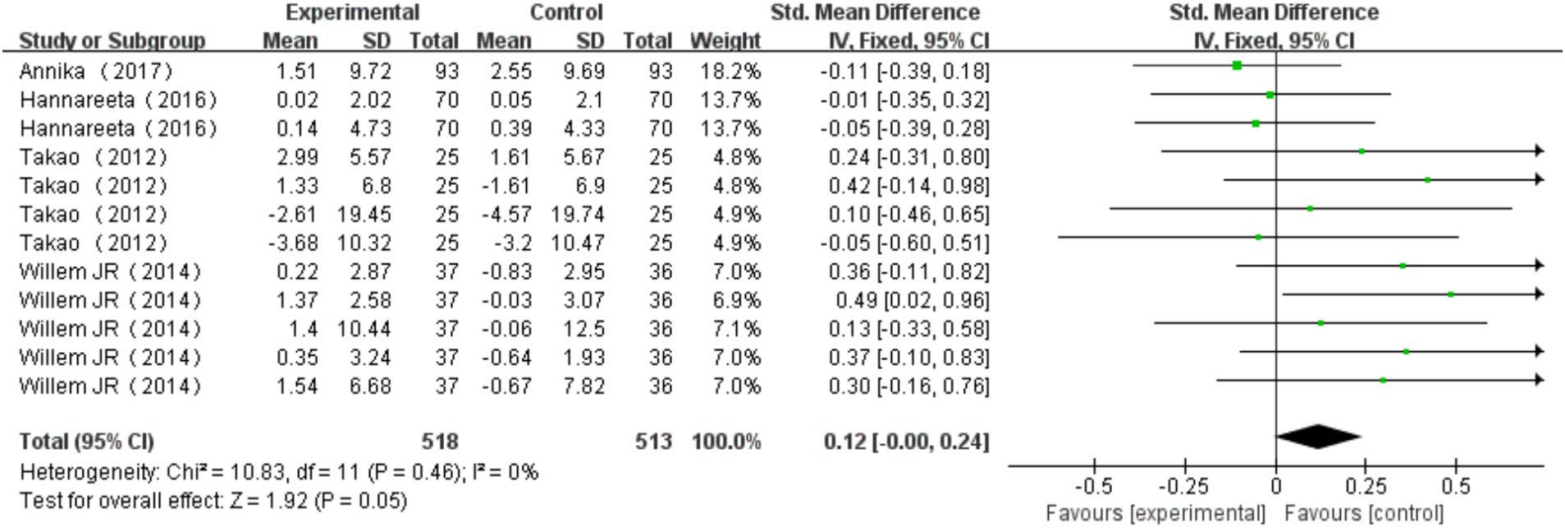
Effect of multi-component exercise on executive function.

A total of 4 studies (Bossers et al., 2015; Öhman et al., 2016; Toots et al., 2017; Suzuki et al., 2012) were included, with one study utilizing two different measurement methods, one study using four different measurement methods, and another employing five different measurement methods. These measurement methods include SCWT-I, SCWT-III, Verbal fluency, Visual memory span backward, Stroop test, Incomplete figures, Digit span backward. Verbal fluency is the most commonly used measurement method. Heterogeneity test results indicated no significant heterogeneity across the studies (*I^2^* = 0%, *p* = 0.46). A fixed effects model was chosen to combine the results, with a combined effect size of SMD = 0.12, 95% CI: −0.00, 0.24, *p* = 0.05. The forest plot illustrated that the impact of multi-component exercise on the cognitive subdomain of executive function in older adults had a 95% CI for SMD that extended to the right of the null line (see Figure 5). The findings suggest that multi-component exercise is effective in improving the cognitive subdomain of executive function in older adults.

##### 3.4.2.3 The effect of multi-component exercise on language

**Figure 6:**
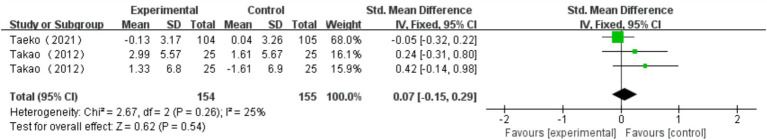
Effect of multi-component exercise on language.

Two studies were included (Suzuki et al., 2012; Makino et al., 2021), with one study employing two different measurement methods. These measurement methods include CVFT, LVFT. Heterogeneity test results indicated no significant heterogeneity among the studies, with *I^2^* = 25% and *p* = 0.26. The fixed effects model was used to combine the results, yielding a pooled effect size of SMD = 0.07 with a 95% CI of −0.15 to 0.29, and *p* = 0.54. The forest plot indicates that the 95% CI of the SMD for the effects of multi-component exercise on the cognitive domain of language in older adults aligns with the null line (see Figure 6). These findings suggest that multi-component exercise has no significant effects on the cognitive domain of language in older adults.

##### 3.4.2.4 The effects of multi-component exercise on memory

**Figure 7:**
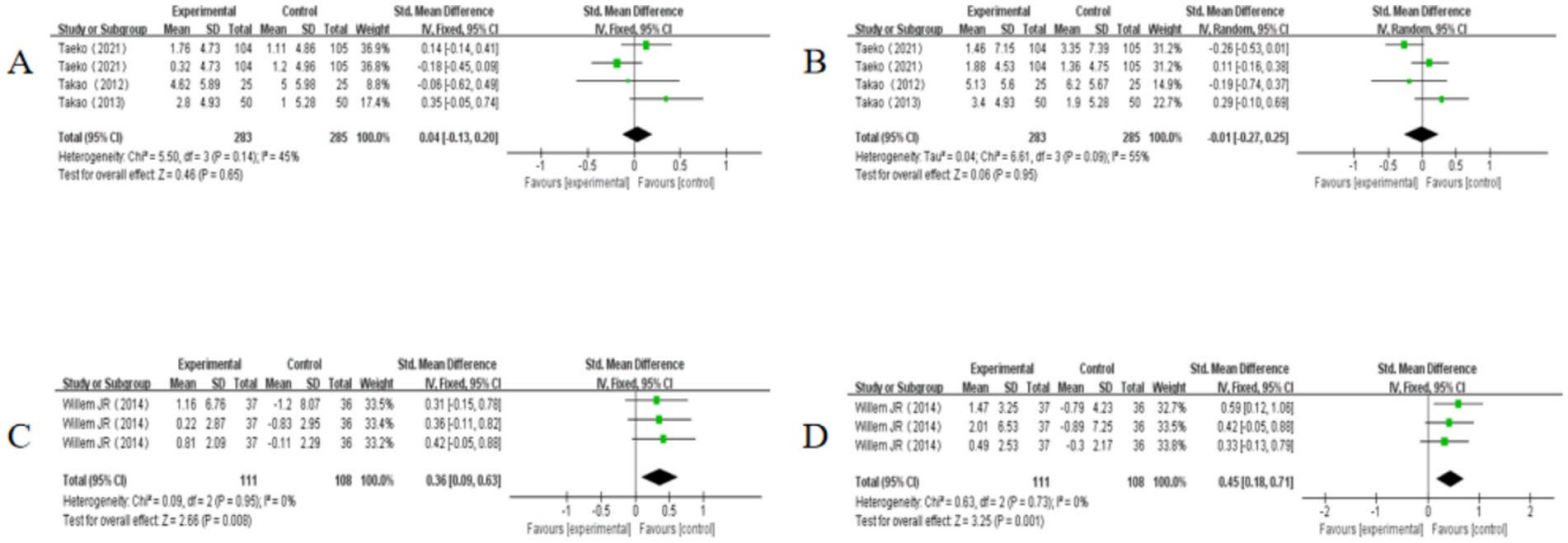
Effect of multi-component exercise on the cognitive subdomain of memory in the elderly. **(A)**: Immediate Memory, **(B)**: Delayed Memory, **(C)**: Verbal Memory, **(D)**: Visual Memory.

In the analysis of immediate memory, three studies (Suzuki et al., 2012; Makino et al., 2021; Suzuki et al., 2013) were included, among which one study utilized two different measurement methods. These measurement methods include WMS-LM I, WMS-R Visual Reproduction I. Examination of heterogeneity revealed no significant differences among the studies, with I^2^ = 45% and *p* = 0.14. Using the fixed effects model to merge the findings, the combined effect size was calculated as SMD = 0.04 with a 95% CI of −0.13 to 0.20 and *p* = 0.65. The forest plot demonstrates that the 95% CI for the impact of multi-component exercise on the cognitive domain of immediate memory in older adults overlaps with the null line (refer to Figure 7A). These outcomes indicate that multi-component exercise does not yield significant effects on the cognitive domain of immediate memory in older adults.

For delayed memory analysis, three studies (Suzuki et al., 2012; Makino et al., 2021; Suzuki et al., 2013) were included, with one study using two different measurement approaches. These measurement methods include WMS-LM II, WMS-R Visual Reproduction II. Heterogeneity analysis indicated noteworthy variations among the studies, with *I^2^* = 55% and *p* = 0.09. Employing the random-effects model to combine the data, the pooled effect size was determined as SMD = −0.01 with a 95% CI of −0.27 to 0.25 and *p* = 0.95. The forest plot illustrates that the 95% CI for the effects of multi-component exercise on the cognitive domain of delayed memory in older adults aligns with the null line (refer to Figure 7B). The findings suggest that multi-component exercise does not have significant effects on the cognitive domain of delayed memory in older adults.

Regarding verbal memory analysis, one study (Bossers et al., 2015) was included, utilizing three different measurement methods. These measurement methods include Digit span forward, 8-Words test–direct recall, 8-Words test–recognition. Heterogeneity testing indicated no substantial differences among the studies, with *I^2^* = 0% and *p* = 0.95. Utilizing the fixed effects model to combine the results, the pooled effect size was calculated as SMD = 0.36 with a 95% CI of 0.09 to 0.63 and *p* = 0.008. The forest plot shows that the 95% CI for the effects of multi-component exercise on the cognitive domain of verbal memory in older adults falls to the right of the null line (refer to Figure 7C). These results suggest that multi-component exercise has significant effects on the cognitive domain of verbal memory in older adults. Lastly, in the examination of visual memory, one study (Bossers et al., 2015) employing three different measurement methods was included. These measurement methods include Faces recognition, Picture recognition, Visual memory span forward. Heterogeneity analysis indicated no significant discrepancies among the studies, with *I^2^* = 0% and *p* = 0.73. By employing the fixed effects model to combine the data, the pooled effect size was computed as SMD = 0.45 with a 95% CI of 0.18 to 0.71 and *p* = 0.001. The forest plot demonstrates that the 95% CI for the effects of multi-component exercise on the cognitive domain of visual memory in older adults falls to the right of the null line (refer to Figure 7D). These findings suggest that multi-component exercise has significant effects on the cognitive domain of visual memory in older adults.

### 3.5 Sensitivity analysis and publication bias

**Figure 8:**
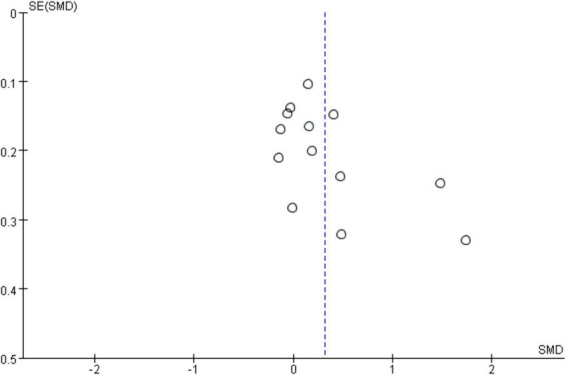
Funnel plot for publication bias.

In the sensitivity analysis, the combined results of the random effects model meta-analysis were consistent with the combined results of the fixed effects model, or when the main results of studies with the lowest quality ratings were excluded, indicating the stability of the synthesized effect (SMD) of exercise interventions on cognitive function. To investigate publication bias, a funnel plot was typically constructed for meta-analyses with more than 10 studies and analyzed. From Figure 8, visual inspection of the funnel plot suggested mild asymmetry, potentially indicating publication bias. Subsequently, an Egger’s test was conducted (Table 2), with a *p*-value of 0.076 > 0.05, indicating no significant publication bias.

**Table 2:** Egger’s test results.

Std_Eff	Cofe	Std. Eff	t	*p* > |t|	(95% Conf. Interval)
Slope	−0.398683	0.3234413	−1.23	0.243	(−1.110572, 0.3132064)
Bias	3.604239	1.840525	1.96	0.076	(−0.4467293, 7.655207)

### 3.6 Subgroup analysis

In order to explore sources of heterogeneity, subgroup analyses were conducted by grouping variables such as exercise frequency, exercise duration, exercise duration and different types of cognitive impairments. The results revealed a decrease in heterogeneity among studies, indicating that exercise frequency, exercise duration, exercise duration, and different types of cognitive impairments may be significant sources of heterogeneity. To further investigate the sources of heterogeneity, sensitivity analyses were conducted by sequentially excluding individual studies included, with results consistent with the original analysis. Individual studies had minimal impact on the pooled results, suggesting the stability of the synthesized effect estimates in this study.

#### 3.6.1 The effect of exercise frequency on overall cognitive function

**Figure 9:**
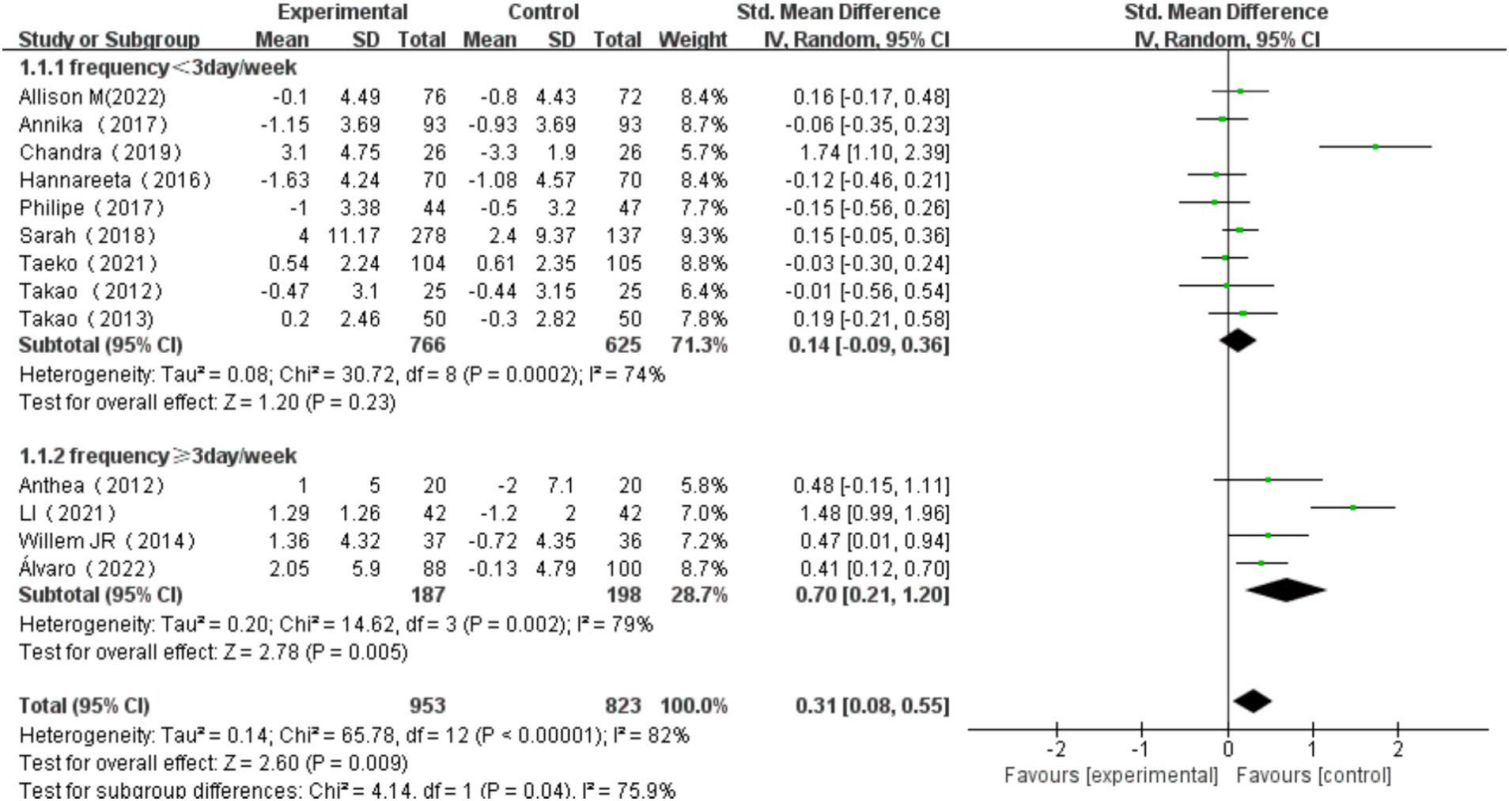
Effect of exercise frequency on overall cognitive function.

All 13 studies were included in this subgroup analysis. Exercise frequency was categorized as <3 days/week and ≥ 3 days/week. For the group exercising <3 days/week, the heterogeneity test yielded χ^2^ = 30.72, *I^2^* = 74%, *p* = 0.0002, with a combined effect size of SMD = 0.14 (95% CI:-0.09, 0.36, *p* = 0.23), indicating no statistically significant difference. For the group exercising≥3 days/week, the heterogeneity test yielded χ^2^ = 14.62, *I^2^* = 79%, *p* = 0.002, with a combined effect size of SMD = 0.70 (95% CI: 0.21, 1.20, *p* = 0.005), indicating a statistically significant difference. Subgroup difference test showed that there was significant heterogeneity between different exercise frequency groups (χ^2^ = 4.14, df = 1, *p* = 0.04, I^2^ = 75.9%).The effect size was most significant for those who exercised≥3 days/week. Additionally, the significant difference in effect size suggests that exercise frequency has a significant effect on cognitive function in older adults (Figure 9).

#### 3.6.2 The effect of intervention length on overall cognitive function

**Figure 10:**
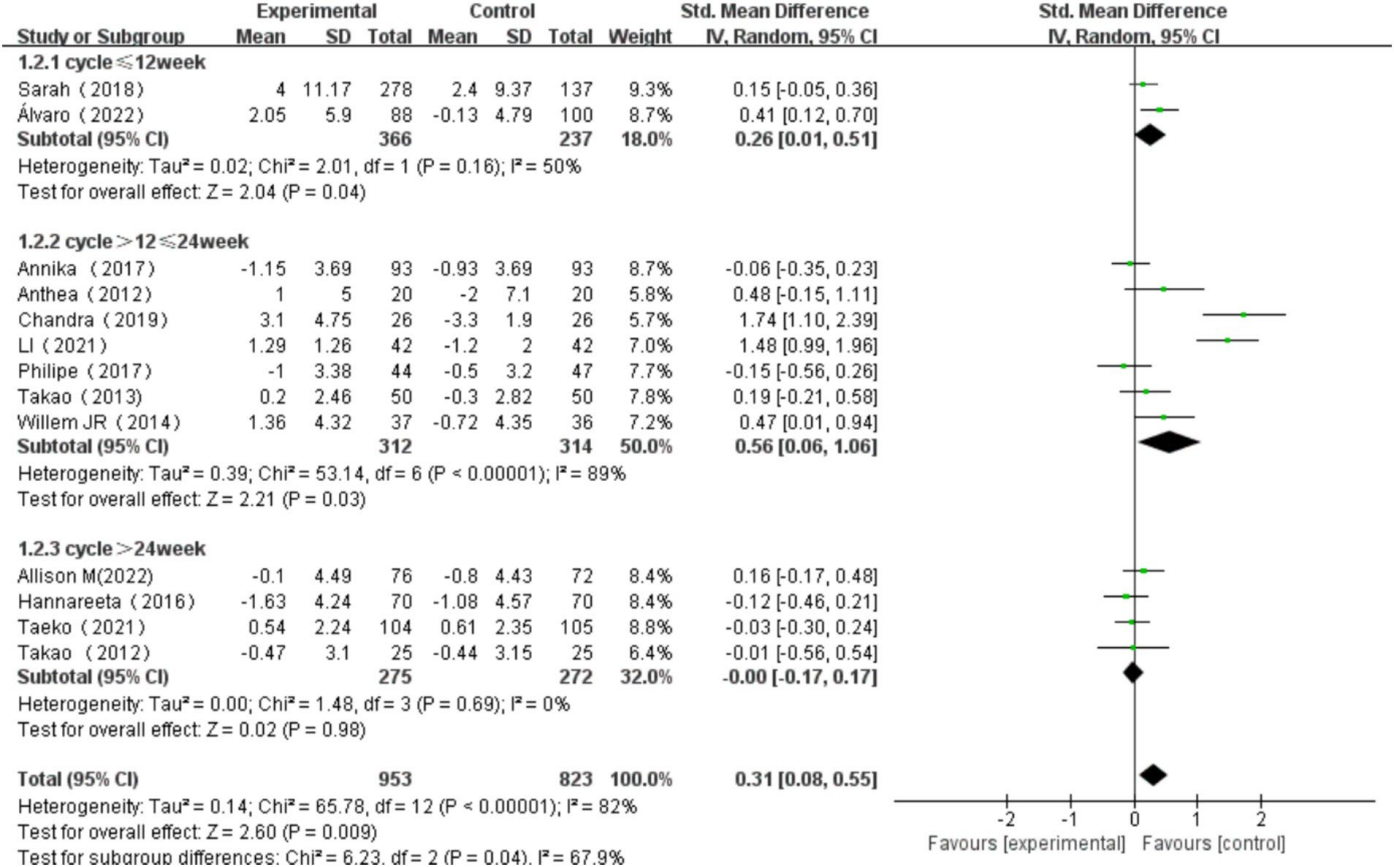
Effect of exercise period on overall cognitive function.

Intervention length was categorized into three groups: ≤12 weeks, >12 weeks and ≤ 24 weeks, and > 24 weeks. For intervention of ≤12 weeks, the heterogeneity test yielded χ^2^ = 2.01, *I^2^* = 50%, *p* = 0.16, with a combined effect size of SMD = 0.26 (95% CI: 0.01, 0.51, *p* = 0.04), indicating a statistically significant difference. For studies that intervened more than >12 weeks and ≤ 24 weeks, the heterogeneity test yielded χ^2^ = 53.14, *I^2^* = 89%, *p* < 0.001, with a combined effect size of SMD = 0.56 (95% CI: 0.06, 1.06, *p* = 0.03), indicating a statistically significant difference. For exercise duration of >24 weeks, the heterogeneity test yielded χ^2^ = 1.48, *I^2^* = 0%, *p* = 0.69, with a combined effect size of SMD = -0.00 (95% CI: −0.17, 0.17, *p* = 0.98), indicating no statistically significant difference. Subgroup difference test showed that there was significant heterogeneity among different exercise period groups (χ^2^ = 6.23, df = 2, *p* = 0.04, I^2^ = 67.9%). The effect size was most significant for exercise duration of >12 and ≤ 24 weeks. These findings suggest that the length of intervention has a significant influence on overall cognitive function in older adults (Figure 10).

#### 3.6.3 The effect of exercise duration on cognitive function

**Figure 11:**
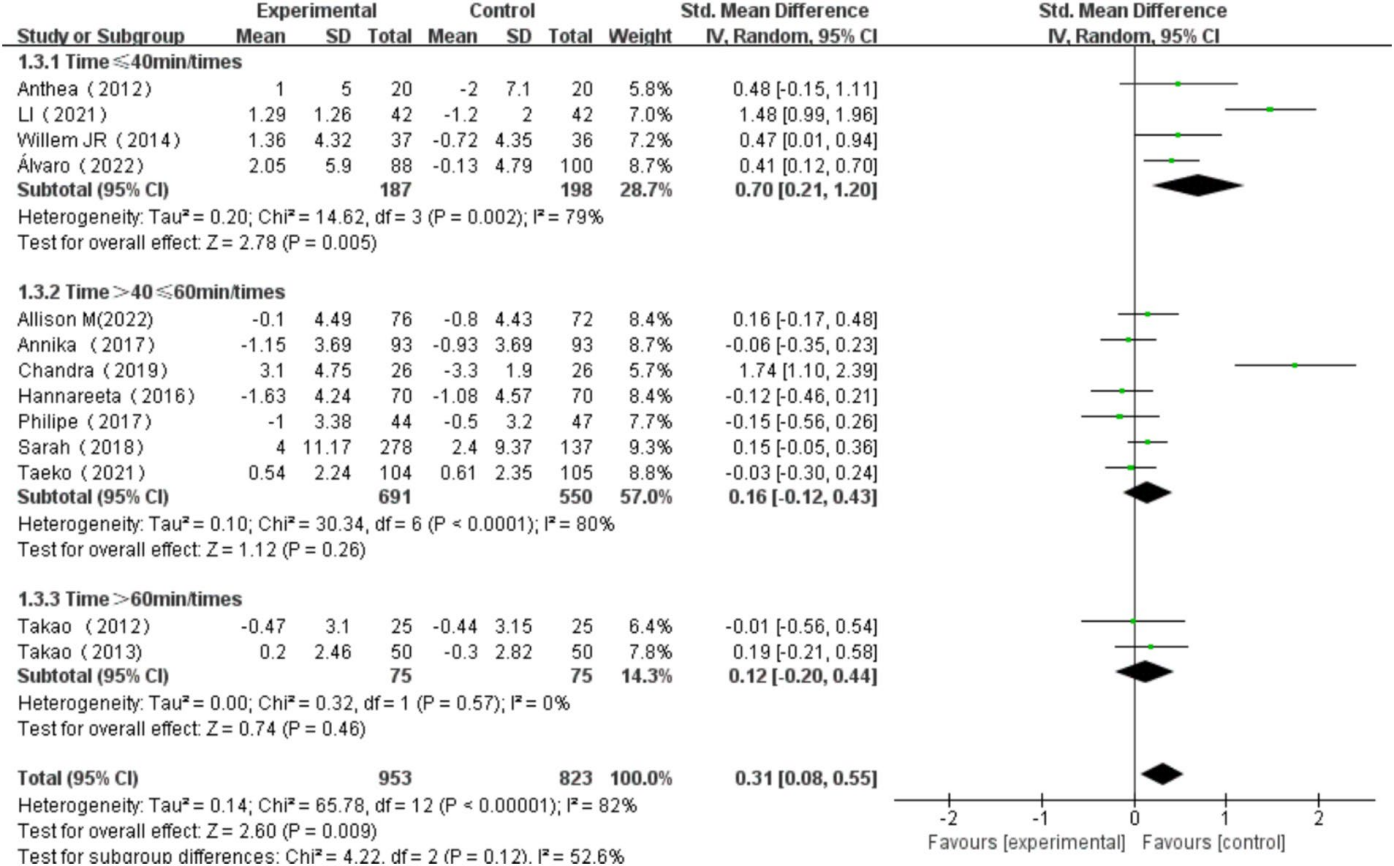
Effect of exercise duration on overall cognitive function.

Exercise duration was categorized into three groups: single exercise session ≤40 min, >40 min and ≤ 60 min, and > 60 min. The heterogeneity test for single exercise duration ≤40 min showed χ^2^ = 14.62, *I^2^* = 79%, *p* = 0.002, with a significant difference in the pooled effect size (SMD = 0.70, 95% CI: 0.21, 1.20, *p* = 0.005). The heterogeneity test for single exercise session>40 min and ≤ 60 min showed χ^2^ = 30.34, *I^2^* = 80%, *p* < 0.001, with no significant difference in the pooled effect size (SMD = 0.16, 95% CI: −0.12, 0.43, *p* = 0.26). The heterogeneity test for single exercise session >60 min showed χ^2^ = 0.32, *I^2^* = 0%, *p* = 0.57, with no significant difference in the pooled effect size (SMD = 0.12, 95% CI: −0.20, 0.44, *p* = 0.46). Among these, the effect size for exercise session of ≤40 min was the most significant (Figure 11). Subgroup difference test showed that there was no significant difference in the effect size between different subgroups (χ^2^ = 4.22, df = 2, *p* = 0.12), but there was moderate heterogeneity (I^2^ = 52.6%). This indicates that subgroup variables may have a certain impact on the intervention effect, but due to sample size limitations or intra-subgroup variation, it does not reach a statistically significant level.

#### 3.6.4 The effects of multi-component exercise on older adults with different types of cognitive impairment

**Figure 12:**
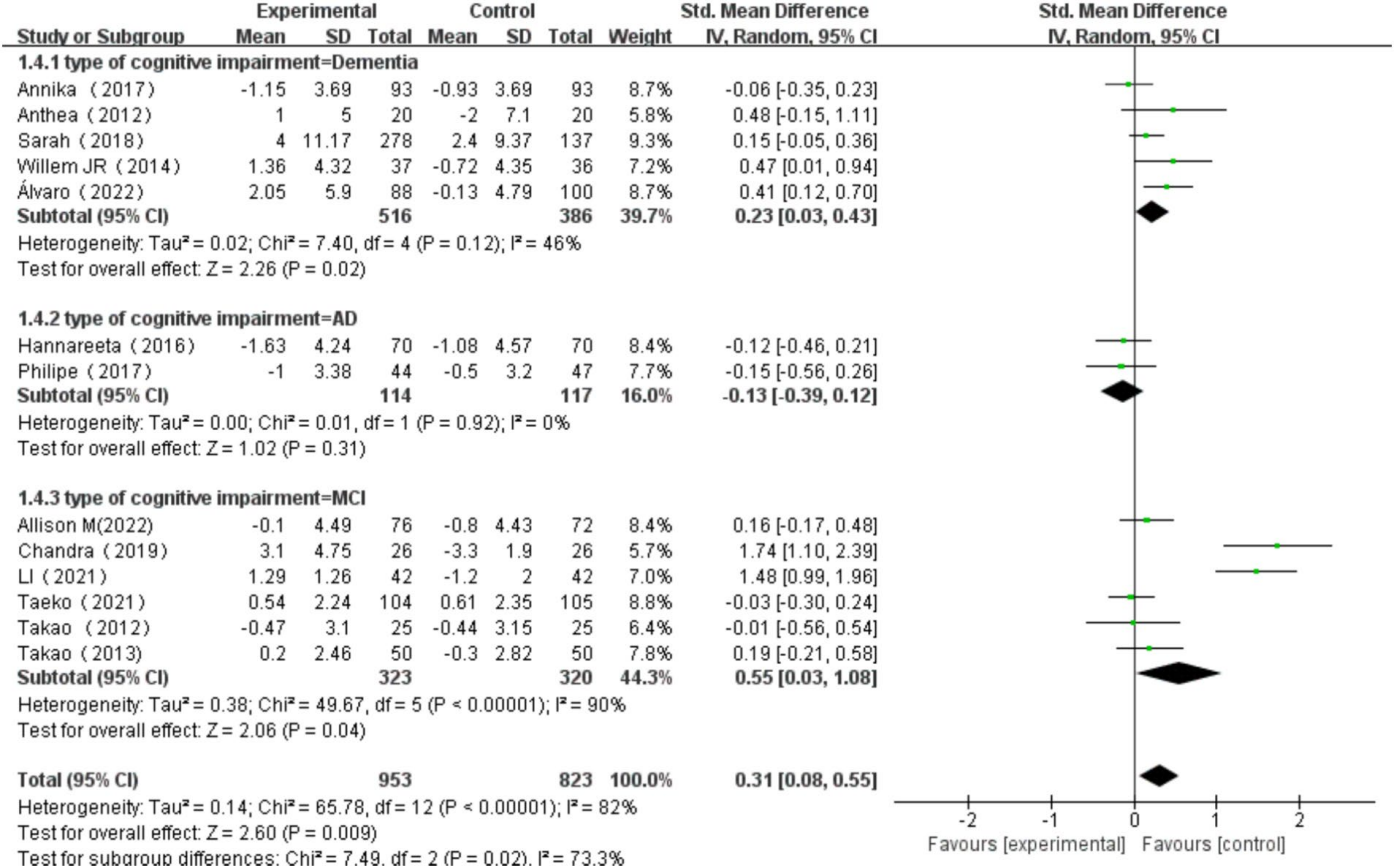
Effect of exercise on cognitive function in elderly individuals with different types of cognitive impairment.

The types of cognitive impairment were categorized into MCI, AD, and dementia (Jongsiriyanyong and Limpawattana, 2018). Heterogeneity tests for dementia patients revealed ^2^ = 7.40, *I^2^* = 46%, p = 0.12, with a significant combined effect size SMD = 0.23, 95% CI: 0.03, 0.43, *p* = 0.02. Heterogeneity tests for AD patients showed χ^2^ = 0.01, *I^2^* = 0%, *p* = 0.92, with a non-significant combined effect size SMD = −0.13, 95% CI: −0.39, 0.12, *p* = 0.31. Heterogeneity tests for MCI patients indicated χ^2^ = 49.67, *I^2^* = 90%, *p* < 0.00001, with a significant combined effect size SMD = 0.55, 95% CI: 0.03, 1.08, *p* = 0.04. Subgroup difference test showed that there was significant heterogeneity among different types of cognitive impairment (χ^2^ = 7.49, df = 2, *p* = 0.02, I^2^ = 73.3%).Among these, the effect size for MCI patients was the most significant (Figure 12).

## 4 Discussion

### 4.1 The effect of multi-component exercise on overall cognitive impairment among older adults

This study represents the first comprehensive analysis demonstrating multi-component exercise’s targeted benefits on executive function and memory through subgroup parameterization, providing an empirical foundation for personalized exercise prescription in cognitive rehabilitation.

As the aging population continues to grow, the prevalence of cognitive impairment in the elderly is also increasing (Nirwan et al., 2020). This trend may be attributed to factors such as frailty, neurodegenerative and vascular diseases, lifestyle, and lack of physical activity (Jongsiriyanyong and Limpawattana, 2018; Rundek et al., 2022; Yaffe et al., 2014). Evidence indicates that multi-component exercise has the potential to elevate the production of neurotrophic factors, such as brain-derived neurotrophic factor (BDNF), in the brain, thereby promoting the growth and preservation of nerve cells. Furthermore, exercise has been shown to promote the development of new blood vessels, improve cerebral blood flow, and increase the supply of oxygen and nutrients to the brain (Chandler et al., 2019). A research study investigating the influence of multi-component exercise on cognitive function in older adults revealed a notable deceleration in overall cognitive decline, with a cumulative effect size of 0.32 (Venegas-Sanabria et al., 2022).

The pathogenesis of cognitive impairment in older adults is intricate, characterized by a decline in cognitive abilities and structural and functional changes in the brain that resemble the pathological basis of neurodegenerative diseases. However, exercise, as a non-pharmacological intervention, has shown effectiveness in improving cognitive function. Multi-component exercise, a comprehensive form of physical activity encompassing aerobic exercise, strength training, balance training, coordination training, and agility training, can enhance brain activation through multiple mechanisms compared to single-component exercise. These mechanisms include increased cerebral blood flow, promotion of neuronal connections, improved brain metabolism, facilitation of interregional connectivity within the brain, and enhancement of neurotransmitter secretion (Li et al., 2021).

These combined mechanisms work together to heighten brain processing speed and thereby improve cognitive function in older individuals. With advancing age, cardiovascular function in elderly individuals naturally declines, leading to a slowdown in cerebral blood circulation (Bertsch et al., 2009). Studies indicate that multi-component exercise can ramp up cerebral blood supply by elevating heart rate and blood pressure, increasing blood flow in the frontal and hippocampal regions, and ultimately improving brain perfusion. This enhanced blood flow can deliver more oxygen and nutrients crucial for executive function, processing speed, and memory, supporting the maintenance and promotion of brain cell metabolism (Hortobágyi et al., 2022). Compared to sedentary older adults, those engaged in long-term exercise exhibit relatively higher cerebral blood flow in regions like the posterior cingulate cortex and the precuneus. However, discontinuation of exercise training may lower cerebral blood flow in brain areas, including the hippocampus (Tarumi and Zhang, 2018). A study by Kaushal et al. (2018) discovered that after a 12-week intervention, multi-component exercise significantly boosted cerebral blood flow and neuroplasticity in older adults compared to pre-intervention levels. Embracing multi-component exercise can effectively drive neurogenesis and in turn enhance cognitive function.

### 4.2 The effects of multi-component exercise on cognitive subdomains in older adults

This meta-analysis demonstrates that multi-component exercise significantly improves executive function and memory in older adults. However, the effects on information processing speed, language, and visuospatial abilities are not significant. This disparity may be attributed to the fact that, as the disease progresses to later stages, brain regions associated with language and memory sustain more severe damage, leading to the collapse of the entire semantic system. Previous research has linked improvements in executive function to neural circuits in the posterior hippocampus, anterior hippocampus, and frontal lobes. These circuits are particularly vulnerable during normal aging and play a critical role in the progression of dementia (Erickson et al., 2011). In the articles included in our analysis (Bossers et al., 2015), a nine-week multi-component exercise regimen induced changes in various brain regions, namely the frontal and hippocampal areas, resulting in cognitive improvements. Enhancements in executive and memory functions were achieved by combining aerobic and strength training exercises. This finding aligns with a 16-week multi-component exercise study conducted on patients with AD (de Andrade et al., 2013). Bossers et al. (2015) found that, compared to a social control group, the multi-component exercise group exhibited significant improvements in cognitive functions, including overall cognition, visual memory, verbal memory, and executive function. Additionally, multi-component exercise (a combination of aerobic and strength training) proved more effective than aerobic exercise alone, in slowing the decline in cognitive and motor functions among dementia patients. This is likely due to the activation of the posterior temporal and prefrontal brain areas associated with cognitive subdomains, thereby improving functions in the executive and memory regions. Strength training tasks that incorporate coordination and balance may also activate cerebellar-cortical connections, positively influencing cognitive subdomains. Bliss et al. (2021) further supports that multi-component exercise significantly enhances executive functions in older women with MCI compared to either aerobic or resistance training alone. Regrettably, this study found no significant effects of multi-component exercise on other cognitive subdomains. The limited number of relevant clinical studies likely accounts for the small number of included studies. Future research should focus on the impact of multi-component exercise on other cognitive subdomains in older adults to strengthen the evidence base.

### 4.3 The moderating effects of multi-component exercise on cognitive function in older adults

Regarding types of cognitive impairment, this meta-analysis found that multi-component exercise had significant effect sizes for both dementia and MCI populations. Notably, the effect size was larger for the MCI group, indicating a more pronounced intervention effect. Aerobic exercise enhances cardiorespiratory fitness and cognitive function, while resistance training improves muscle strength and retains daily living capabilities. Additionally, balance and coordination training help improve daily functioning and reduce the risk of falls. By leveraging the strengths of these various exercise forms, multi-component exercise can more comprehensively improve both cognitive function and physical fitness in individuals with MCI. Compared to single-mode interventions, multi-component exercise can more effectively address the complex cognitive challenges of MCI patients by stimulating both mind and body, thereby improving memory-related and frontal lobe-related cognitive functions (Yang et al., 2021). Engaging in multiple types of exercise helps increase cardiac output, enhancing cerebral blood circulation and augmenting the supply of oxygen and nutrients. This, in turn, promotes the metabolism and repair of neural cells, thereby effectively improving cognitive function in patients with dementia (Richard and Ligthart, 2010). Furthermore, multi-component exercise can enhance brain structural plasticity and potentially improve executive function in AD patients by stimulating sensorimotor adaptation and promoting the coordination of variable fiber bundles (Lv et al., 2023). Unfortunately, this study found that the effects of multi-component exercise on the AD population were not significant. By the clinical stages of AD, the brain may have endured substantial damage, limiting the efficacy of exercise in improving cognitive function. At this stage of the disease, the potential benefits of exercise may no longer be fully realized (Öhman et al., 2016). However, the limited number of included studies is a constraint, highlighting the need for more clinical research on the impact of multi-component exercise on cognitive function in AD patients. Future research should focus on increasing the evidence base to enable a more robust evaluation of this intervention.

Exercise frequency, duration, and length of intervention were the main sources of heterogeneity among the included studies. In terms of exercise frequency, the largest effect sizes were observed with exercise sessions occurring three or more times per week. A meta-analysis indicated that engaging in exercise 5 to 7 times per week yielded the optimal cognitive function improvement for adults over the age of 50 (Northey et al., 2018). Furthermore, studies have shown that various types of exercise training, performed 5 to 7 times per week, had a greater impact on cognitive function compared to exercising 2 times per week or less (Quigley et al., 2020). This is likely due to the fact that high-frequency exercise can enhance the formation and reorganization of neural connections, upregulate levels of neurotrophic factors such as BDNF and IGF-1, lower inflammation levels, reduce brain damage, and consequently improve cognitive function. Regarding exercise duration, sessions lasting 40 min or less showed the most significant effect sizes. The included studies in this meta-analysis had interventions with a maximum exercise duration of 120 min. Prolonged single exercise sessions may lead to fatigue and affect the effectiveness and motivation of older adults for exercise participation. Shorter exercise durations, on the other hand, may be more easily accepted by older adults as they perceive them to be manageable and sustainable, thus enhancing their motivation to engage in physical activity. Hence, selecting exercise sessions within a time frame of 40 min or less is deemed more appropriate. Regarding the intervention period, exercise interventions lasting 3 to 6 months yielded the largest effect sizes and demonstrated the best outcomes. It has been found that due to factors such as neurodegenerative changes, shorter intervention periods may fail to achieve ideal intervention effects (Guo et al., 2022). Conversely, longer intervention periods may lead to adaptive responses by the body to the consistent exercise regimen and, in conjunction with physiological factors stemming from neurodegenerative changes, exercise fatigue, risk of injury, and limited adaptability, gradually diminish the intervention effects. These findings underscore the need for a more comprehensive understanding of the impact of neurodegenerative changes on exercise interventions, in order to develop more effective strategies. It should be noted that the combination mode, cycling method, and intensity differences in multi-component exercise training may also be significant sources of heterogeneity among the included studies. However, the existing research is insufficient to differentiate the effects of different combinations and sequences of exercise types on intervention outcomes. As for intensity, while the majority of studies included in this meta-analysis positioned it at a moderate level, there is inconsistency in the criteria used by the various studies to define exercise intensity. Future research should prioritize standardized intensity indicators such as heart rate reserve (e.g., %HRmax) or Borg’s Rating of Perceived Exertion (RPE) scale. For instance, moderate intensity could be defined as 64–76% of HRmax or RPE 12–13, while vigorous intensity as>77% HRmax or RPE ≥ 14–17. Such standardization would enhance the operationalizability of exercise prescriptions for cognitive rehabilitation.

Although the present study strictly adhered to the PRISMA checklist, the following limitations and constraints should be acknowledged: (1) The literature search did not include unpublished literature, and some articles were not included due to lack of complete outcome data, potentially impacting the comprehensiveness of the data; (2) Inconsistencies existed among the study participants, such as age, gender, and severity of illness, which may have affected result accuracy; (3) Some studies did not use blinding or did not explicitly specify whether a concealed allocation method was used, which may have increased bias risk; (4) The intervention period varied from 9 to 48 weeks, with most studies centered around 12 to 24 weeks. Currently, research on the long-term effects of exercise interventions on cognitive function in older adults remains limited, and this study did not analyze the longest exercise intervention period in multi-component exercise interventions for cognitive function in older adults; (5) The inclusion of multiple outcomes from single studies may underestimate heterogeneity and inflate the weight of individual studies. Future meta-analyses should employ hierarchical models or composite scores to mitigate this bias; (6)Furthermore, the time interval between our literature search cutoff (January 2024) and submission may have excluded newly published studies, potentially affecting the comprehensiveness of the evidence; (7) due to the small sample size of subgroup analysis and no multiple test correction, the differences between different subgroups need to be carefully interpreted.

## 5 Conclusion

The results of this meta-analysis indicate that multi-component exercise interventions can effectively delay the decline in cognitive function in older adults, particularly in executive function and memory subdomains, with the intervention effects being modulated by various variables. In terms of exercise intervention variables, the most effective intervention was found to be multi-component exercise performed for less than or equal to 40 min per session, 3 times or more per week, for a duration of 3 to 6 months. For older adults with different types of cognitive impairment, multi-component exercise interventions were found to be most effective for those with MCI. However, due to limited studies and significant heterogeneity, these findings should be interpreted with caution. To provide further evidence, more RCTs with standardized study designs are needed. In addition, there is a lack of research focused on the effects of multi-component exercise interventions on specific cognitive domains in older adults, which should be a priority for future research.

## Data Availability

The original contributions presented in the study are included in the article/Supplementary material, further inquiries can be directed to the corresponding authors.

## References

[ref1] Bertsch K. Hagemann D. Hermes M. Walter C. Khan R. Naumann E. (2009). Resting cerebral blood flow, attention, and aging. Brain Res. 1267, 77–88. doi: 10.1016/j.brainres.2009.02.053, PMID: 19272361

[ref2] Bliss E. S. Wong R. H. Howe P. R. Mills D. E. (2021). Benefits of exercise training on cerebrovascular and cognitive function in ageing. J. cereb. Blood Flow Metab. 41, 447–470. doi: 10.1177/0271678X20957807, PMID: 32954902 PMC7907999

[ref3] Blomstrand P. Tesan D. Nylander E. M. Ramstrand N. (2023). Mind body exercise improves cognitive function more than aerobic- and resistance exercise in healthy adults aged 55 years and older - an umbrella review. Euro. Rev. Aging Phys. Act. 20:15. doi: 10.1186/s11556-023-00325-4, PMID: 37558977 PMC10413530

[ref4] Bossers W. J. van der Woude L. H. Boersma F. Hortobágyi T. Scherder E. J. van Heuvelen M. J. (2015). A 9-week aerobic and strength training program improves cognitive and motor function in patients with dementia: a randomized, controlled trial. Am. J. Geriatr. Psychiatry 23, 1106–1116. doi: 10.1016/j.jagp.2014.12.191, PMID: 25648055

[ref5] Casas-Herrero Á. Sáez de Asteasu M. L. Antón-Rodrigo I. Sánchez-Sánchez J. L. Montero-Odasso M. Marín-Epelde I. . (2022). Effects of Vivifrail multicomponent intervention on functional capacity: a multicentre, randomized controlled trial. J. Cachexia. Sarcopenia Muscle 13, 884–893. doi: 10.1002/jcsm.12925, PMID: 35150086 PMC8977963

[ref6] Chandler M. J. Locke D. E. Crook J. E. Fields J. A. Ball C. T. Phatak V. S. . (2019). Comparative effectiveness of behavioral interventions on quality of life for older adults with mild cognitive impairment: a randomized clinical trial. JAMA Netw. Open 2:e193016. doi: 10.1001/jamanetworkopen.2019.3016, PMID: 31099860 PMC6537922

[ref7] Cordes T. Bischoff L. L. Schoene D. Schott N. Voelcker-Rehage C. Meixner C. . (2019). A multicomponent exercise intervention to improve physical functioning, cognition and psychosocial well-being in elderly nursing home residents: a study protocol of a randomized controlled trial in the PROCARE (prevention and occupational health in long-term care) project. BMC Geriatr. 19:369. doi: 10.1186/s12877-019-1386-6, PMID: 31870314 PMC6929376

[ref8] Cress M. E. Buchner D. M. Prohaska T. Rimmer J. Brown M. Macera C. . (2005). Best practices for physical activity programs and behavior counseling in older adult populations. J. Aging Phys. Act. 13, 61–74. doi: 10.1123/japa.13.1.61, PMID: 15677836

[ref9] de Andrade L. P. Gobbi L. T. Coelho F. G. Christofoletti G. Costa J. L. Stella F. (2013). Benefits of multimodal exercise intervention for postural control and frontal cognitive functions in individuals with Alzheimer's disease: a controlled trial. J. Am. Geriatr. Soc. 61, 1919–1926. doi: 10.1111/jgs.12531, PMID: 24219193

[ref10] de Souto B. P. Cesari M. Denormandie P. Armaingaud D. Vellas B. Rolland Y. (2017). Exercise or social intervention for nursing home residents with dementia: a pilot randomized, controlled trial. J. Am. Geriatr. Soc. 65, E123–e129. doi: 10.1111/jgs.14947, PMID: 28542742

[ref11] Du Z. Li Y. Li J. Zhou C. Li F. Yang X. (2018). Physical activity can improve cognition in patients with Alzheimer's disease: a systematic review and meta-analysis of randomized controlled trials. Clin. Interv. Aging 13, 1593–1603. doi: 10.2147/CIA.S169565, PMID: 30233156 PMC6130261

[ref12] Erickson K. I. Voss M. W. Prakash R. S. Basak C. Szabo A. Chaddock L. . (2011). Exercise training increases size of hippocampus and improves memory. Proc. Natl. Acad. Sci. USA 108, 3017–3022. doi: 10.1073/pnas.1015950108, PMID: 21282661 PMC3041121

[ref13] Espeland M. A. Lipska K. Miller M. E. Rushing J. Cohen R. A. Verghese J. . (2017). Effects of physical activity intervention on physical and cognitive function in sedentary adults with and without diabetes. J. Gerontol. A Biol. Sci. Med. Sci. 72, 861–866. doi: 10.1093/gerona/glw179, PMID: 27590629 PMC6075086

[ref14] Guo L. Liu Z. Yuan W. (2022). The effect of Baduanjin on the balancing ability of older adults: a systematic review and meta-analysis. Front. Med. 9:995577. doi: 10.3389/fmed.2022.995577, PMID: 36388883 PMC9650403

[ref15] Higgins J. P. Altman D. G. Gøtzsche P. C. Jüni P. Moher D. Oxman A. D. . (2011). The Cochrane Collaboration's tool for assessing risk of bias in randomised trials. BMJ 343:d5928. doi: 10.1136/bmj.d5928, PMID: 22008217 PMC3196245

[ref16] Hortobágyi T. Vetrovsky T. Balbim G. M. Sorte Silva N. C. B. Manca A. Deriu F. . (2022). The impact of aerobic and resistance training intensity on markers of neuroplasticity in health and disease. Ageing Res. Rev. 80:101698. doi: 10.1016/j.arr.2022.101698, PMID: 35853549

[ref17] Jongsiriyanyong S. Limpawattana P. (2018). Mild cognitive impairment in clinical practice: a review article. Am. J. Alzheimers Dis. Other Dement. 33, 500–507. doi: 10.1177/1533317518791401, PMID: 30068225 PMC10852498

[ref18] Kaushal N. Desjardins-Crépeau L. Langlois F. Bherer L. (2018). The effects of multi-component exercise training on cognitive functioning and health-related quality of life in older adults. Int. J. Behav. Med. 25, 617–625. doi: 10.1007/s12529-018-9733-0, PMID: 29926316

[ref19] Lamb S. E. Sheehan B. Atherton N. Nichols V. Collins H. Mistry D. . (2018). Dementia and physical activity (DAPA) trial of moderate to high intensity exercise training for people with dementia: randomised controlled trial. BMJ 361:k1675. doi: 10.1136/bmj.k1675, PMID: 29769247 PMC5953238

[ref20] Langoni C. D. S. Resende T. L. Barcellos A. B. Cecchele B. Knob M. S. Silva T. D. N. . (2019). Effect of exercise on cognition, conditioning, muscle endurance, and balance in older adults with mild cognitive impairment: a randomized controlled trial. J. Geriatr. Phys. Ther. 42, E15–e22. doi: 10.1519/JPT.0000000000000191 29738405

[ref21] Li L. Liu M. Zeng H. Pan L. (2021). Multi-component exercise training improves the physical and cognitive function of the elderly with mild cognitive impairment: a six-month randomized controlled trial. Ann. Palliat. Med. 10, 8919–8929. doi: 10.21037/apm-21-1809, PMID: 34488379

[ref22] Lv S. Wang Q. Liu W. Zhang X. Cui M. Li X. . (2023). Comparison of various exercise interventions on cognitive function in Alzheimer's patients: a network meta-analysis. Arch. Gerontol. Geriatr. 115:105113. doi: 10.1016/j.archger.2023.105113, PMID: 37418819

[ref23] Mak A. Delbaere K. Refshauge K. Henwood T. Goodall S. Clemson L. . (2022). Sunbeam program reduces rate of falls in long-term care residents with mild to moderate cognitive impairment or dementia: subgroup analysis of a cluster randomized controlled trial. J. Am. Med. Dir. Assoc. 23, 743–749.e1. doi: 10.1016/j.jamda.2022.01.064, PMID: 35196481

[ref24] Makino T. Umegaki H. Ando M. Cheng X. W. Ishida K. Akima H. . (2021). Effects of aerobic, resistance, or combined exercise training among older adults with subjective memory complaints: a randomized controlled trial. J. Alzheimers Dis. 82, 701–717. doi: 10.3233/JAD-210047, PMID: 34092635

[ref25] Moher D. Liberati A. Tetzlaff J. Altman D. G. (2009). Preferred reporting items for systematic reviews and meta-analyses: the PRISMA statement. PLoS Med. 6:e1000097. doi: 10.1371/journal.pmed.1000097, PMID: 19621072 PMC2707599

[ref26] Nirwan J. S. Hasan S. S. Babar Z. U. Conway B. R. Ghori M. U. (2020). Global prevalence and risk factors of gastro-oesophageal reflux disease (GORD): systematic review with Meta-analysis. Sci. Rep. 10:5814. doi: 10.1038/s41598-020-62795-1, PMID: 32242117 PMC7118109

[ref27] Northey J. M. Cherbuin N. Pumpa K. L. Smee D. J. Rattray B. (2018). Exercise interventions for cognitive function in adults older than 50: a systematic review with meta-analysis. Br. J. Sports Med. 52, 154–160. doi: 10.1136/bjsports-2016-096587, PMID: 28438770

[ref28] Öhman H. Savikko N. Strandberg T. E. Kautiainen H. Raivio M. M. Laakkonen M. L. . (2016). Effects of exercise on cognition: the Finnish Alzheimer disease exercise trial: a randomized, controlled trial. J. Am. Geriatr. Soc. 64, 731–738. doi: 10.1111/jgs.14059, PMID: 27037872

[ref29] Quigley A. MacKay-Lyons M. Eskes G. (2020). Effects of exercise on cognitive performance in older adults: a narrative review of the evidence, possible biological mechanisms, and recommendations for exercise prescription. J. Aging Res. 2020, 1–15. doi: 10.1155/2020/1407896, PMID: 32509348 PMC7244966

[ref30] RichardE. LigthartS. A. (2010). Moll van Charante EP, van Gool WA: methodological issues in a cluster-randomized trial to prevent dementia by intensive vascular care. J. Nutr. Health Aging 14, 315–317. doi: 10.1007/s12603-010-0072-3, PMID: 20306005

[ref31] Rudnicka E. Napierała P. Podfigurna A. Męczekalski B. Smolarczyk R. Grymowicz M. (2020). The World Health Organization (WHO) approach to healthy ageing. Maturitas 139, 6–11. doi: 10.1016/j.maturitas.2020.05.018, PMID: 32747042 PMC7250103

[ref32] Rundek T. Tolea M. Ariko T. Fagerli E. A. Camargo C. J. (2022). Vascular Cognitive Impairment (VCI). Neurotherapeutics 19, 68–88. doi: 10.1007/s13311-021-01170-y, PMID: 34939171 PMC9130444

[ref33] Sadjapong U. Yodkeeree S. Sungkarat S. Siviroj P. (2020). Multicomponent exercise program reduces frailty and inflammatory biomarkers and improves physical performance in community-dwelling older adults: a randomized controlled trial. Int. J. Environ. Res. Public Health 17:760. doi: 10.3390/ijerph17113760, PMID: 32466446 PMC7312630

[ref34] Shah H. Albanese E. Duggan C. Rudan I. Langa K. M. Carrillo M. C. . (2016). Research priorities to reduce the global burden of dementia by 2025. Lancet Neurol 15, 1285–1294. doi: 10.1016/S1474-4422(16)30235-6, PMID: 27751558

[ref35] Song D. Yu D. S. F. Li P. W. C. Lei Y. (2018). The effectiveness of physical exercise on cognitive and psychological outcomes in individuals with mild cognitive impairment: a systematic review and meta-analysis. Int. J. Nurs. Stud. 79, 155–164. doi: 10.1016/j.ijnurstu.2018.01.002, PMID: 29334638

[ref36] Suzuki T. Shimada H. Makizako H. Doi T. Yoshida D. Ito K. . (2013). A randomized controlled trial of multicomponent exercise in older adults with mild cognitive impairment. PLoS One 8:e61483. doi: 10.1371/journal.pone.0061483, PMID: 23585901 PMC3621765

[ref37] Suzuki T. Shimada H. Makizako H. Doi T. Yoshida D. Tsutsumimoto K. . (2012). Effects of multicomponent exercise on cognitive function in older adults with amnestic mild cognitive impairment: a randomized controlled trial. BMC Neurol. 12:128. doi: 10.1186/1471-2377-12-128, PMID: 23113898 PMC3534485

[ref38] Tabloski P. A. (2004). Global aging: implications for women and women's health. JOGNN 33, 627–638. doi: 10.1177/0884217504268655, PMID: 15495709

[ref39] Tarumi T. Zhang R. (2018). Cerebral blood flow in normal aging adults: cardiovascular determinants, clinical implications, and aerobic fitness. J. Neurochem. 144, 595–608. doi: 10.1111/jnc.14234, PMID: 28986925 PMC5874160

[ref40] Tisher A. Salardini A. (2019). A comprehensive update on treatment of dementia. Semin. Neurol. 39, 167–178. doi: 10.1055/s-0039-1683408, PMID: 30925610

[ref41] Toots A. Littbrand H. Boström G. Hörnsten C. Holmberg H. Lundin-Olsson L. . (2017). Effects of exercise on cognitive function in older people with dementia: a randomized controlled trial. J. Alzheimers Dis. 60, 323–332. doi: 10.3233/JAD-170014, PMID: 28800328 PMC5611799

[ref42] Vaughan S. Wallis M. Polit D. Steele M. Shum D. Morris N. (2014). The effects of multimodal exercise on cognitive and physical functioning and brain-derived neurotrophic factor in older women: a randomised controlled trial. Age Ageing 43, 623–629. doi: 10.1093/ageing/afu010, PMID: 24554791

[ref43] Venegas-Sanabria L. C. Cavero-Redondo I. Martínez-Vizcaino V. Cano-Gutierrez C. A. Álvarez-Bueno C. (2022). Effect of multicomponent exercise in cognitive impairment: a systematic review and meta-analysis. BMC Geriatr. 22:617. doi: 10.1186/s12877-022-03302-1, PMID: 35879665 PMC9316334

[ref44] Vreugdenhil A. Cannell J. Davies A. Razay G. (2012). A community-based exercise programme to improve functional ability in people with Alzheimer's disease: a randomized controlled trial. Scand. J. Caring Sci. 26, 12–19. doi: 10.1111/j.1471-6712.2011.00895.x, PMID: 21564154

[ref45] Wang X. Wang H. Ye Z. Ding G. Li F. Ma J. . (2020). The neurocognitive and BDNF changes of multicomponent exercise for community-dwelling older adults with mild cognitive impairment or dementia: a systematic review and meta-analysis. Aging 12, 4907–4917. doi: 10.18632/aging.102918, PMID: 32191630 PMC7138588

[ref46] Yaffe K. Falvey C. M. Hoang T. (2014). Connections between sleep and cognition in older adults. Lancet Neurol. 13, 1017–1028. doi: 10.1016/S1474-4422(14)70172-3, PMID: 25231524

[ref47] Yang S. Y. Lee H. C. Huang C. M. Chen J. J. (2021). Efficacy of tai chi-style multi-component exercise on frontal-related cognition and physical health in elderly with amnestic mild cognitive impairment. Front. Aging 2:636390. doi: 10.3389/fragi.2021.636390, PMID: 35822039 PMC9261301

[ref48] Zheng G. Xia R. Zhou W. Tao J. Chen L. (2016). Aerobic exercise ameliorates cognitive function in older adults with mild cognitive impairment: a systematic review and meta-analysis of randomised controlled trials. Br. J. Sports Med. 50, 1443–1450. doi: 10.1136/bjsports-2015-095699, PMID: 27095745

